# Measuring Outcomes of Psychological Well-Being within Paediatric Health Settings

**DOI:** 10.3390/healthcare6010001

**Published:** 2017-12-29

**Authors:** Halina Flannery, Sarah Glew, Aylana Brewster, Deborah Christie

**Affiliations:** University College London Hospital, Child and Adolescent Psychological Services, 6th Floor, 250 Euston Road, NW1 2PG London, UK; sarah.glew@cantab.net (S.G.); ay-aylana@hotmail.co.uk (A.B.)

**Keywords:** patient-reported outcome measures, paediatric, emotional well-being, psychological well-being, quality of life, narratives

## Abstract

There are many widely used, validated patient reported outcome measures for physical and mental health. However, capturing outcomes from young people living with chronic health conditions presents a challenge, needing to take the complex interplay of physical and mental health into consideration. The authors found that regularly used outcome measures for child and adolescent emotional well-being in paediatric settings largely fall into two groups: paediatric quality of life and child and adolescent mental health measures. The applicability and potential for use of these measures within this context is discussed. Whilst offering some utility, neither approach leaves sufficient space for young people’s individual experiences of illness and treatment. The scope of using alternative qualitative approaches that capture the rich stories and experiences of young people living with chronic illnesses is considered.

## 1. Introduction

The psychological impact of chronic health conditions for young people, the importance of integrating psychological and physical care, and the necessity to support holistic health outcomes have been highlighted by many [1,2,3].

Many outcome measures collected by paediatric healthcare professionals as part of routine practice are used for service improvement and clinical learning, and are regularly driven by management and government, to compare the quality and effectiveness of interventions and evaluate cost-effectiveness [4]. Outcome measures in healthcare have traditionally focused on measures of mortality and on the associated physiological markers such as blood pressure and test results but, with the increase in patient autonomy and choice, have increasingly included aspects of patient experience of perceived outcomes [5].

The shift away from a reliance on physiological health outcomes has led to frequent measurement of functional status, health perception, and preference-based measures as well as patient-reported experience measures (PREMs) and patient-reported outcome measures (PROMs). PREMs focus on aspects of satisfaction with patient experience, such as waiting times and clinical communication, and will not be discussed further here.

The current paper reviews current PROMs and discusses their validity and utility in a paediatric psychology setting. It concludes with a description of a narrative approach to outcome measurement, its potential for capturing meaningful outcomes, and suggestions for future work.

## 2. Patient Reported Outcomes Measures—PROMs

The use of PROMs is widespread in paediatric healthcare settings. Many of the measures have evolved from means to measure effectiveness in clinical trials, but now are used to measure functional status and wellbeing, according to patients’ own reports, during their healthcare journey [6]. Both condition-specific and generic PROMs have been developed, with the potential to improve service-delivery and enable comparisons of healthcare provisions that can guide patient decision in elective procedures [7]. Though there is little research on the effectiveness of using PROMs in improving healthcare, Valderas and colleagues [8] express ‘optimism’ for the effectiveness of their use, and the necessity of more methodologically sound research to assess this. The use of PROMs has many challenges including ensuring and retaining patient participation, measuring meaningful aspects of care, interpreting results, overcoming issues with standardised questionnaires, and matching best outcomes measures to the population [7,9].

### Psychological Well-Being, Chronic Health Conditions, and PROMs

The impact of living with chronic health conditions on the psychological well-being of children and young people is widely documented, as is the imperative to collect PROMs that measure this [10]. However, a literature search, carried out to identify PROMs of psychological well-being for young people living with chronic health conditions, revealed no clear consensus or recommendations on best practice to do this (A search spanned Google Scholar and psychological databases using keywords including ‘outcome measures’, ‘PROMs’ ‘child’, ‘adolescent’, ‘well-being’, and ‘quality of life’. In addition, an extensive grey literature search was conducted, including all relevant measures on the Child Outcomes Research Consortium (CORC) website (CORC, 2017).)

Validated and widely used PROMs for physical health and mental health exist but a challenge is presented in how to capture outcomes in a meaningful way that takes into consideration the complex interplay of physical and mental health, in a context of childhood development, when living with a chronic health condition [11]. Regularly used outcome measures for child and adolescent emotional well-being largely fall into two groups: paediatric quality of life and child and adolescent mental health. The utility, applicability, and limitations of these two groups of measures for capturing psychological well-being in paediatric health services is considered and an alternative, qualitative approach to capturing meaningful outcomes is also presented.

## 3. Quality of Life PROMs

Quality of Life (QoL) is defined as “physical and emotional well-being, level of independence, social relationships, and their relationship to salient features of their environment” [12] (p. 1405), a concept capturing both emotional and physical well-being [13]. However, discrepancies in definition of quality of life prove problematic [14]. Despite a broad consensus that emotional well-being is constituent to quality of life conceptually, many of the tools identified are primarily focused on health-related quality of life, not adequately capturing psychological well-being. Many tools use ‘functional status’ and ‘health status’ interchangeably with ‘quality of life’ and so investigate only symptom management and adaptive functioning [15]. Although these aspects are undoubtedly important to psychological well-being, they cannot capture young people’s wider emotional experience. This conflation of quality of life with health functional status makes it more difficult to find an appropriate measure to thoroughly investigate emotional wellbeing in this population.

A number of PROMs are explicitly ‘health-related quality of life’ (HQoL) measures, and these include the KINDL^R^ Questionnaire for Measuring Health-Related Quality of Life in Children and Adolescents, Revised Version (KINDL^R^) [16], the Pediatric Quality of Life Inventory (PedsQL) [17], Dimension Health-Related Quality of Life (16D) [18], and the RAND Health Status Measure for Children (HSMC) [19]. Wallander, Schmitt, and Koot [14] argue that this term produces a false dichotomy between ‘health-related quality of life’ and a quality of life that is somehow not health-related. The authors powerfully state: “QL (quality of life) is by its nature a holistic concept … How can one’s life be separated into what is influenced by a disease from that which is influenced by all current and past experiences” (p. 435).

HQoL PROMs may inadequately pick up other aspects of quality of life in this population, reinforcing a young person’s identity as a patient, rather than honouring their rich identity and the levels of well-being therein. Young people with a chronic illness view themselves and their lives in the same way as peers who are healthy [20], so the outcomes that are important to them are likely to be similar to those measured in typical populations.

Although all QoL measures identified contain sections or individual items looking at socio-emotional markers of quality of life, in the majority of available measures, these items are limited in number and depth. For example, the Dartmouth COOP Functional Health Assessment Charts [21] include a scale on emotional feelings, but one question asks, ‘during the past month, how often did you feel anxious, depressed, irritable, sad or downhearted and blue?’ and is scored on a 5-point Likert-type scale, not allowing separation of worry from low mood. The Impact of Weight Quality of Life Kids scale [22] includes scales on body esteem, social life, and family relations but does not contain any other items relating to emotional well-being more widely. The use of single domains in isolation is likely to lack psychometric validity. Some measures act as short indexes examining overall functioning, with a few questions on wellbeing contributing to a total index score. For example, the Quality of Wellbeing Scale [23] provides a short measure of functional status designed to help calculate Quality of Life Years. While useful for cost-effectiveness calculations, these PROMs have limited utility as indicators of emotional wellbeing in young people with chronic health conditions. Their scope is limited by a small number of prescriptive possible answers within each domain and total index scores may be more influenced by the domain of physical health.

Some HQoL measures provide more detailed sections on emotional well-being and on social and functional status in addition to more health-related items. The PedsQL [17] is a widely used measure that includes short sections on emotional, social, and school functioning, which can be combined as a Psychosocial Health Summary Score, in addition to a Physical Health Summary Score. Modules are also available for specific conditions (including arthritis, diabetes, cancer, asthma, cerebral palsy, muscular dystrophy, eosinophilic esophagitis, gastrointestinal symptoms, multidimensional fatigue, pediatric pain, and sickle cell disease) and contain sections about condition-specific worries and the experience of communicating with doctors. Similarly, the KINDL^R^ [16], despite looking at HQoL, was designed for both ‘clinical’ and ‘healthy’ populations and contains short sections on general feelings, feelings about self, relationships with friends, family, and school alongside a section on physical health, and has condition-specific modules (including adiposity, asthma, diabetes, epilepsy, neurodermitis, oncology and spina bifida) and a version for prolonged stays in hospital. The items can be used as sub-scales (emotional well-being, physical well-being, self-esteem, school, friendships, and family) that have reasonable psychometric properties [16].

Many questionnaires do not extend below 5 or 6 years old, the youngest age at which a consensus holds that children can reliably self-report aspects of their quality of life or wellbeing [24]. Below this age, a small number of parent report measures are available (e.g., Infant and Toddler Quality of Life Questionnaire, [25]) though this proxy measure introduces bias, with parent and child responses often varying widely [26]. The Pictured Child’s Quality of Life Self Questionnaire has been developed in France and validated for ages 4–11 years (and a separate questionnaire for adolescents) and, although translated, does not yet appear to be widely used in the UK [27]. One group has developed an innovative board game that facilitates the collection of qualitative information about life satisfaction in pre-school-aged children with a chronic illness, overcoming some of the limitations of language and cognition encountered when deriving outcomes from younger children (Satisfaction in Life for Children with Own Report Measures (SILCWORM) [28]). The authors identified three outcome themes that are covered by the PedsQL measure (physical, emotional, and social functioning) and six additional themes (family, feeling and worries, daily routine, coping, medical treatment, and disclosure), which the children report to have an important impact on quality of life and emotional well-being and are less commonly covered in QoL questionnaires.

There are a large number of QoL and HQoL measures used with children with chronic illness. However, these often focus on health-specific and symptom-based outcomes or produce a general index score and are not specialised for children’s age. These measures are also rarely designed for routine outcome collection and so may not capture change over time. There is a lack of psychological outcome measures specifically designed to be used in physical health, with generic measures such as the PedsQL and the KINDL currently providing the most extensive and age specific coverage of psychological outcomes but not leaving enough space for young people’s individual experiences of illness and treatment or to collect the outcomes young people report as meaningful.

## 4. Mental Health PROMs for Children and Adolescents

Community Children and Adolescents’ Mental Health Services (CAMHS) across the UK have increasingly been under pressure to routinely collect outcomes of mental health and well-being in young people in order to provide evidence-based, effective, and patient-centred services [29,30,31]. As a result, consensus on best practice for how to measure PROMs within child mental health services is emerging from national collaborations, such as the Child Outcomes Research Consortium (CORC), and government initiatives, such as Children and Young People’s Improving Access Psychological Therapy (CYP-IAPT). CYP-IAPT was developed as a service transformation model for CAMHS with the fundamental aim of improving the quality of services. An underpinning value is ‘accountability’, which is expressed by rigorous and routine monitoring of outcomes [32]. Physical illness is considered a risk factor for developing mental health difficulties [33], and paediatric psychology services often have strong links with or are embedded within CAMHS. Although not all paediatric health services will be directly affected by CAMHS mandatory outcome collection, the principles underpinning the collection of PROMs are relevant and there is a similar imperative to measure outcome measures of emotional well-being in paediatric settings [10]. The applicability of the recommended outcome measures for child mental health services to young people with chronic health conditions is considered.

Recommended outcome measures are categorised into those with a broad focus, such as the Strength and Difficulties Questionnaire (SDQ) [34], The Revised Children’s Anxiety and Depression Scale [35], and Goal-Based Outcomes [36], and those with a focus specific for presentations, populations, and relationships, such as the RCADS subscales for social phobia, panic, and OCD, the Systemic Clinical Outcome and Routine Evaluation (SCORE-15) tool for use in family therapy [37], the Eating Disorders Examination Questionnaire [38], and the Sheffield Learning Disabilities Outcome Measure [39]. Some of the recommended measures are clinician-scored therapeutic alliance measures, or PREMs, and so are beyond the scope of this review, but may be useful for those intentions. The measures are predominantly focused on symptoms of mental health difficulties, including anxiety, depression, and eating disorders, which may be useful for assessment and monitoring of young people experiencing more severe psychological difficulties. As with QoL PROMs, the majority of these measures can also not be completed directly by the youngest children in services, limiting their utility.

Although paediatric and CAMHS psychological services may often be linked and may overlap in their remits, the nature of referrals varies greatly. CAMHS services are most likely to receive referrals for young people with acute and chronic mental health difficulties, whereas paediatric services will work with young people living with a chronic illness. Their experience of this may impact their psychological well-being, challenging self-identity and relationships and inducing health-related worry and low mood. Conversely, their psychological well-being may impact their health; a young person’s frustration with living with their condition may impact adherence to treatment. The recommended mental health measures are not designed to capture the subtle impact on emotional well-being that young people often experience in living with chronic illnesses and are therefore unlikely to be fit for purpose in paediatric settings. The frustration, anger, and sadness of having to look after diabetes every day may not amount to ‘depression’ but will impact a young person’s psychological well-being and may get in the way of their social and academic functioning. Furthermore, in some cases, the symptoms of ‘mental health disorders’ might overlap with symptoms of the chronic illness. Items from the SDQ and RCADS relating to fatigue, hyperactivity, and irritability (“I get a lot of headaches, stomach aches and sickness”, “I am easily distracted, I find it difficult to concentrate”, “I have trouble sleeping”, “I have problems with my appetite”, “I have no energy for things”, and “I think about death”) overlap with symptoms of hypo- and hyperglycaemia, other commonly experienced symptoms of chronic health conditions and treatment effects of chemotherapy. Caution must therefore be taken in the interpretation of these measures.

Some of the more individualised outcome measures such as Goal Based Outcomes [36] may be more applicable although they are lacking in psychometric rigour. They can be more individualised to measure outcomes of importance to young people and their families by allowing a practitioner and young person to collaboratively set goals and rate them on a scale from 1 to 10. The Child Outcome Rating Scale [40], which uses scales for a young person to rate their overall well-being and their well-being at home, at school, and with friends, could provide a brief tool to consider the emotional well-being of a young person living with chronic illness and the potential impact of chronic illness on different areas of their life [41]. However, these measures may be too narrow and miss the breadth and complexity of the experience of a young person living with chronic illness.

In summary, routine PROMs of mental health may be relevant to screen and monitor for mental health difficulties in young people with chronic illness who are experiencing higher levels of distress (with caution taken in the interpretation of particular items). However, they may be less sensitive to the nuances and impact of living with chronic illness. Currently, there is no specific focus on chronic health conditions and the interplay with psychological well-being.

## 5. Moving towards Narratives—How to Capture the ‘Full Story’

Currently available outcome measures sit on two ends of a spectrum, with mental health PROMs focusing on specific mental health difficulties without accessing the nuance of distress encountered in chronic illness and QoL measures focusing too heavily on health-specific and symptom-based outcomes. Neither approach leaves sufficient space for young people’s individual experiences of illness and treatment. However, the service pressure to use outcome measures to demonstrate service effectiveness and understand what is meaningful to young people remains [10]. Quantitative measures may appear to more easily assess effectiveness, but should not be the only approach to understanding outcomes of importance to young people and their families. Merlino and Raman [42] argue that reaching out to service commissioners in a humanitarian way and moving away from predominantly objective and numerical approaches will allow connection with patients with caregivers, fostering improved patient experience and service delivery and providing world-class care. They highlight the importance of ‘stories’ in building understanding and connection in order to improve service delivery. Giving service users the opportunity to explain their experiences in a health service and of living with a chronic illness, without specific predefined questions, adds depth and attempts to understand what users expect from a service. One study that used focus groups to discuss outcome measures with young CAMHS users found that clients feel the need for something more ‘sophisticated to supplement a simple “tick-box” approach’ and suggested that the ‘opportunity to capture something yourself and talk’ might be helpful [43]. In addition, quantitative methods rarely sufficiently take into account the age of children. In order to collect meaningful outcomes in children and young people with chronic illness, measures should be tailored to an appropriate age range because child development presents a rapidly changing context, progresses at different rates for different children and is markedly different from adulthood, with children uniquely embedded in a series of social contexts (the family, school, and peers) [11,24]. Context may affect how children experience a chronic illness.

Qualitative approaches to understanding young people and their families’ experience of services may begin to fill this gap. Girling et al. [44] worked with young people with type 1 diabetes to design an experience measure (PREM) that would capture the aspects of clinical experience important to them. While crucial to healthcare delivery, this does not allow for adequate measurement of wellbeing outcomes.

The child and adolescent psychology service at University College London Hospital (UCLH) has also faced the difficulty of identifying a single measure that was relevant for the number of long term conditions seen in the service (including medically unexplained symptoms, diabetes, cancer, epilepsy, gastroenterological disorders, and rheumatological conditions). Referrals vary from non-adherence to treatment, managing a terminal condition and managing pain, fatigue, frustration, and anger as well as anxiety or condition-related low mood and distress. None of the quantitative measures available adequately captured the outcomes of importance for the young people seen or were a good fit with the narrative ethos and practices of the team. Given this challenge, the team adopted a pragmatic approach to the collection of outcomes with the intention of capturing the stories and outcomes that young people and their families identify as important, without using a questionnaire. Verbatim outcomes from young people were collected in psychological therapy sessions at different points of intervention and analysed to identify the recurrent themes of important outcomes. The themes appeared to cut across the conditions and included managing the health condition (including their relationship with the health condition and approaches to managing symptoms and treatment), managing emotions (including sadness, anger, anxiety, and guilt), finding space to talk, communication and relationships (particularly within families about the health condition), and adaptive functioning and social connectedness (including attendance at school and engagement in social activities and friendships). This systematic but less structured method demonstrates great potential describing psychological outcomes in paediatric settings. It provides a richness of data that contributes to an understanding of the young people in the service, opens up new narratives between young clients and clinicians, and encourages reflexivity amongst clinicians and service managers. The emerging themes from the qualitative outcomes include areas that are not adequately captured by existing structured outcome measures.

The current analysis did not explicitly include looking at age or other demographic factors of the young people in developing the themes. These factors are rich, important parts of the context around young people’s medical journey and so are likely to have a large effect on the outcomes that are most meaningful to them. However, the richness of data collected and method of inductive analysis utilised allows for further examination, including a look into particular outcomes by diagnosis, gender, and age.

The team recognises that this qualitative approach to collecting outcomes makes it difficult to monitor change over time and does not fit easily within a numbers-driven health care system. Collecting and analysing this kind of data in a narrative way is also more time-consuming and demands more resources than numerical analysis, which may pose a particular difficulty to services under financial pressure.

The richness of capturing the stories of young people’s journeys with chronic health conditions is strongly in line with agendas for patient-centred care and can prove to be a powerful tool to connect service providers and commissioners to service users [42].

## 6. Where Do We Go from Here?

Traditional quantitative measures are too broad and miss the rich description of young people’s experience of emotional well-being in physical health settings. More idiographic measures may be too narrow and lacking in psychometric rigour. New methods of collecting qualitative data through narrative approaches, such as gathering verbatim feedback, co-authoring measures with young people and families, or standardising conversations by means of a game, offer potential avenues for making outcomes more meaningful. At present, collecting meaningful outcomes from young people within paediatric health settings while meeting the demands of service providers to ‘evidence’ good practice and cost-effectiveness remains a challenge. There is a lack of validated and applicable outcome measures to be used to these ends, so more validated tools could be developed in the future to enable healthcare professionals to monitor and measure outcomes of well-being in this population. However, if services and commissioners really wish to know the most important outcomes to young people in paediatric settings, and indeed to any ages in any settings, the answer may not be to develop more measurement tools; a sea change in outcome research and measurement may be necessary. Capturing the rich stories and self-reported experiences of young people, free from the constraints of quantitative, payment-by-results paradigms, and sharing this with service providers and clinicians will lead to more effective services in the eyes of service-users [42]. Furthermore, these methods and measures must be utilised outside the domain of psychology services and become part of routine practice for entire multi-disciplinary teams. The psychological wellbeing, quality of life, and potential mental health difficulties of young people must all be looked after by the entire medical team.
